# Mobile Laminar Airflow for Intravitreal Injections: Reducing Microbial Load at the Instrument Field

**DOI:** 10.3390/jcm15062362

**Published:** 2026-03-19

**Authors:** Vittoria Satriani, Giovanni Boccia, Biagio Santella, Ferdinando Cione, Antonio Donato, Emanuela Santoro, Aldo De Rosa, Maddalena De Bernardo, Nicola Rosa

**Affiliations:** 1 Department of Medicine, Surgery and Dentistry “Scuola Medica Salernitana”, University of Salerno, 84081 Salerno, Italy; vsatriani@unisa.it (V.S.); gboccia@unisa.it (G.B.); bsantella@unisa.it (B.S.); adonato@unisa.it (A.D.); esantoro@unisa.it (E.S.); nrosa@unisa.it (N.R.); 2 DAI Department of Health Hygiene and Evaluative Medicine, A.O.U. San Giovanni di Dio e Ruggi d’Aragona, 84131 Salerno, Italy; 3 U.O.C. Hospital Hygiene and Laboratory Services, A.O.U. San Giovanni di Dio e Ruggi d’Aragona, 84131 Salerno, Italy; 4 University Eye Unit, A.O.U. San Giovanni di Dio e Ruggi d’Aragona, 84131 Salerno, Italy; fcione@unisa.it (F.C.); alderosa@unisa.it (A.D.R.)

**Keywords:** intravitreal injection, laminar airflow, environmental monitoring, airborne contamination, endophthalmitis prevention

## Abstract

**Background/Objectives**: Intravitreal injections (IVIs) are increasingly performed in outpatient settings, raising concerns regarding how to guarantee operating-theatre-level environmental safety. Mobile laminar airflow (LAF) units may create an ultraclean instrument field, but microbiological evidence from real-world IVI clinics is limited. **Methods**: We performed environmental monitoring during three IVI sessions, each including approximately 20 injections per session, in an outpatient procedure room equipped with a mobile LAF device (Operio Toul Mobile). Airborne microbial contamination was measured with a SAS Super 100 impactor (1 m^3^ per sample) at two locations, the procedure-room air and the LAF field, across seven predefined time points (T_−1_to T_5_). Surface contamination of the instrument-covering drape was assessed at mid- and end-session using 24 cm^2^ contact plates on four culture media. Colonies were expressed as CFU/m^3^ or CFU/24 cm^2^ and analysed using a two-way repeated-measures ANOVA (location × time), with Holm-adjusted within-session paired post hoc comparisons at each time point. **Results**: During LAF operation (T_0_–T_4_), mean airborne load was 89.8 ± 10.8 CFU/m^3^ in room air versus 10.9 ± 4.6 CFU/m^3^ under LAF, corresponding to an 87.9% mean reduction (Holm-adjusted *p* < 0.01). At T_−1_ and T_5_ (LAF off), counts were not significantly different between locations. Airborne microbial species consisted mainly of skin/oral commensals; no obligate pathogens were detected. All 24 drape samples showed 0 CFU. **Conclusions**: In this high-throughput outpatient IVI clinic, the mobile LAF device maintained a stable ultraclean microenvironment at the instrument field despite moderate background room contamination, supporting its use as an adjunct to standard aseptic measures, without the need to change the covering drape during the session.

## 1. Introduction

Intravitreal injections (IVIs) are among the most frequently performed ophthalmic procedures worldwide and represent a cornerstone in the management of many retinal diseases, including neovascular age-related macular degeneration, diabetic macular edema, macular edema secondary to retinal vein occlusion, and several inflammatory conditions [1]. By delivering therapeutic agents directly into the vitreous cavity, IVIs allow high intraocular drug concentrations with limited systemic exposure and have substantially improved the visual prognosis and quality of life for large numbers of patients [2,3]. As the population ages and the burden of chronic retinal diseases increases, the number of IVIs performed each year continues to rise, and the procedure has progressively shifted toward high-throughput outpatient pathways [4].

Although IVIs are brief, minimally invasive procedures usually performed under topical anesthesia, they are not risk-free. The most feared complication is post-injection endophthalmitis, a rare but potentially sight-threatening intraocular infection [5]. Other complications include intraocular hemorrhage, retinal detachment, and transient or sustained increases in intraocular pressure. Overall, the safety profile of IVIs is favorable and well documented; however, the very high procedural volume means that even low complication rates have important clinical, organizational, and economic implications [6,7]. In this context, optimizing infection prevention and control (IPC) around IVI delivery is a priority for healthcare systems.

A key unresolved issue concerns the most appropriate clinical setting for performing IVIs, particularly whether these procedures should be conducted in conventional operating theatres or in outpatient procedure rooms that follow standardized aseptic protocols [8]. Recommendations and regulations vary considerably between countries and institutions: in some healthcare systems, IVIs are mandated to be performed in conventional operating theatres, whereas in others they are routinely carried out in procedure rooms or office-based environments that apply local aseptic protocols [8]. This heterogeneity reflects an ongoing debate on how best to balance accessibility, efficiency, and infection control for high-volume ophthalmic procedures. Ensuring environmental safety in outpatient IVI rooms is particularly relevant where demand is high and access to conventional operating theatres is limited [9].

Modern operating theatres are typically equipped with engineered ventilation systems designed to limit airborne microbial contamination of the surgical field, such as unidirectional laminar airflow (LAF) systems [10]. In contrast, outpatient procedure rooms often rely on standard ventilation and simple aseptic precautions. To bridge this gap, mobile LAF units have been introduced or reintroduced into various surgical settings, including ophthalmology [11]. These devices are engineered to create a localized ISO 5-equivalent ultraclean microenvironment within standard clinical rooms by delivering a continuous, unidirectional airflow over the operative field and instrument table. Through high-efficiency H14 HEPA filtration and horizontal laminar airflow distribution, LAF units effectively reduce airborne particle counts and microbial contamination in the protected zone by displacing and diluting surrounding room air [12].

The Operio Toul Mobile integrates these technological features into a compact, mobile platform that can be positioned close to the procedural area, providing targeted environmental control without the structural requirements of a conventional operating room.

Evidence from orthopedic and ophthalmic settings suggests that mobile LAF units can markedly reduce colony-forming unit (CFU) counts at the operative site, even in rooms that do not meet traditional operating theatre standards [12]. However, environmental microbiological data specifically focused on IVI sessions in outpatient procedure rooms remain limited. In particular, the extent to which mobile LAF can maintain a persistently low microbial burden at the instrument field throughout high-throughput IVI lists is not fully characterized [13]. Moreover, there is a need to better understand the relationship between room-wide contamination and the microenvironment directly surrounding the instruments and injection area, where the risk of inoculating microorganisms into the eye is highest.

In this context, the Ophthalmology Department of the “San Giovanni di Dio e Ruggi d’Aragona” University Hospital in Salerno reorganized its outpatient intravitreal service by converting a clean procedure room into a setting suitable for high-volume IVIs and equipping it with a mobile LAF unit. The overarching goal was to combine the logistical advantages of an outpatient pathway—short waiting times, reduced length of stay, and optimized patient flow—with enhanced environmental microbiological control at the operative site.

The primary aim of the present study was to evaluate the environmental safety of this setup by systematically assessing the effectiveness of the LAF device (Operio Toul Mobile) in reducing microbial load at the operative site compared with the surrounding procedure-room air during outpatient IVI sessions. Additionally, the study sought to determine whether mobile LAF can maintain an ultraclean microenvironment around the instrument field throughout real-world, high-volume outpatient IVI sessions by assessing surface contamination of the instrument-covering drape.

## 2. Materials and Methods

This study was performed in the Eye Unit of the “San Giovanni di Dio e Ruggi d’Aragona” University Hospital in Salerno between September 2024 and November 2025.

Fifty-eight adult patients, among three IVIs sessions, aged between 45 and 92 years, 34 females and 24 males (41.4% male) were considered for the study, including patients scheduled to receive IVIs with steroids or anti-VEGF agents with diabetic maculopathy, neovascular age-related macular degeneration, myopic choroidal neovascularization, or macular edema.

Three days before the IVI, patients followed a pre-operative home-based prophylaxis treatment with diclofenac sodium 0.1% (Visunac, Visufarma, Rome, Italy) and chlorhexidine gluconate (Dropsept, Fidia, Abano Terme, Italy). Before administering the IVI, patients were subjected to standard preparation for intraocular surgery with povidone–iodine 0.5% and anesthetic eye drops. Once the procedure is over (each surgical procedure takes 10 to 15 min, with a turnover time of 3 to 5 min between patients) the patients are accompanied outside the room and prepared to go home along with chloramphenicol eye drops prescription to be administered three times a day for five days.

### 2.1. Laminar Airflow Device

The Operio Toul Mobile (Normeditec/Toul Meditech, Sissa Trecasali, Italy) unidirectional LAF unit, which delivers a horizontal ultraclean airflow directly over the surgical field and the instrument table, was used for peri-operative protection. The device is equipped with an H14 HEPA filter that works with the Most Penetrating Particle Size (MPPS) method (>99.95% removal of particles ≥0.3 µm). The instrument supplies a 400–600 m^3^/h of air through a hood that generates the airflow in a column at a speed of 0.4–0.5 m/s, achieving particle levels consistent with ISO 5 over the protected zone.

### 2.2. Air Sampling

The airborne microbial load was assessed using a portable sampler (SAS Super 100 microbial air sampler VWR International S.r.l., Milan, Italy), a device widely employed for environmental monitoring in hospital and cleanroom settings. For each sampling point, the instrument aspirated air at a fixed, calibrated flow rate of 100 L/min for 10 min, corresponding to a total sampled volume of 1000 L (1 m^3^) per sample. Calibration was verified according to the manufacturer’s specifications prior to the study period.

Two different sampling locations were defined a priori with precise spatial references: OS (Operative Site) and DOS (Distant Operative Site). The Operative Site (OS) sampling point was positioned immediately adjacent to the mobile LAF unit, within the airflow field generated by the device and next to the surgical field. The air sampler inlet was positioned at a standardized height of approximately 1 m above the floor, corresponding to the level of the operative field. The Distant Operative Site (DOS) sampling point was positioned at the same OS sampling standardized height, 1 m distant from the mobile LAF unit and adjacent to the operating bed.

Sampling was conducted across IVI sessions with varying patient volumes to assess how effectively the laminar airflow (LAF) system maintained low contamination levels at the instrument table as surgical throughput increased. Environmental measurements were conducted during three IVI sessions, each including approximately 20 injections, with individual procedures lasting about 10–15 min.

Airborne microbial contamination was assessed at seven predefined time points (T_−1_, T_0_, T_1_, T_2_, T_3_, T_4_, T_5_) during each IVI session. At T_−1_, sampling was performed in the Distant Operative Site (DOS) before the start of the IVI list and before activation of the mobile laminar airflow (LAF) unit, representing the baseline background condition. T_0_ measurements were carried out immediately after the Operio Toul Mobile LAF device was powered on, both in the DOS and in the Operative Site (OS), before patient preparation and before the first IVI. T_1_, T_2_, T_3_, and T_4_ samples were collected hourly by clock time (i.e., in 60-min intervals), in both DOS and OS, under full operative conditions with ongoing injections. T_5_ measurements were obtained at the end of the IVI session in both DOS and OS, immediately after completion of the last injection and with the Operio Toul Mobile unit switched off, in order to characterize end-of-session contamination.

### 2.3. Instrument-Covering Drape Sampling

Surface sampling of the instrument-covering drape was conducted at two time points per session (mid-session, T_2_, and end of session, T_5_) using 24 cm^2^ contact plates.

For each sampling time point, four contact plates were used for surface sampling, one for each culture medium: Plate Count Agar (PCA) for total CFU counts, MacConkey agar for Gram-negative bacteria, Baird-Parker agar for *Staphylococcus* spp., and Sabouraud dextrose agar for yeasts, in accordance with standard laboratory protocols. Results were expressed as CFU per 24 cm^2^. Sampling was performed on a critical surface, namely the no-touch area of the sterile cover drape (“filter drape”) protecting the instrument table under laminar airflow, in order to assess whether sterility was maintained throughout the entire surgical list.

### 2.4. Microbiological Processing and Identification

**Figure 1:**
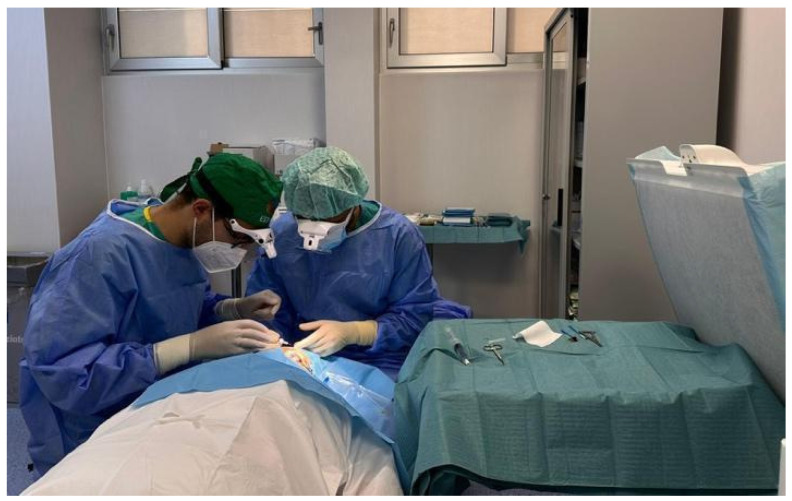
Photographic representation taken during intravitreal injection. The photo shows that the operative site is in front of the laminar airflow.

**Figure 2:**
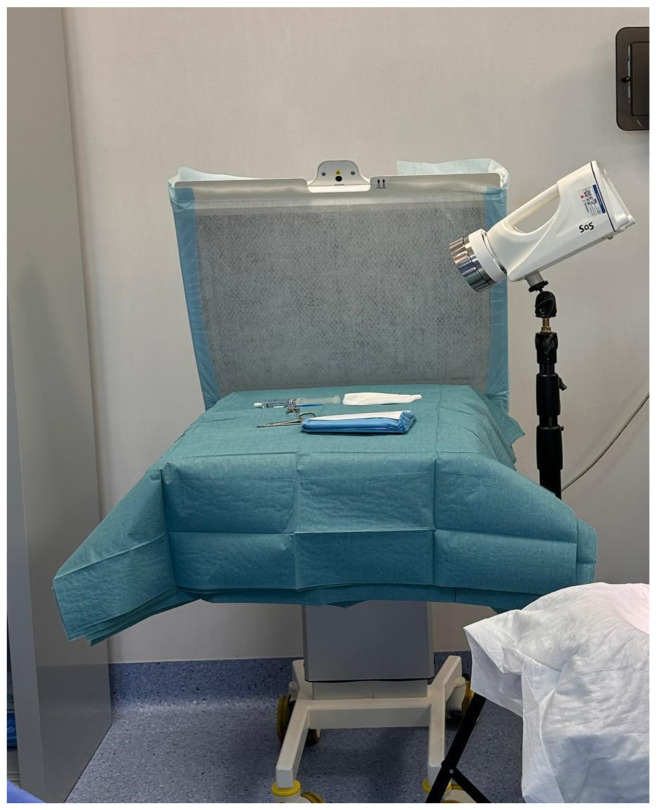
The SAS device is positioned within the laminar airflow for air sampling. The photo shows the filter drape protecting the instrument area under laminar airflow.

The air stream was directed through a perforated sampling head onto 24 cm^2^ Petri dishes containing Plate Count Agar (PCA), where microorganisms were impacted onto the agar surface. Following each sampling cycle, plates were incubated for 24–48 h at 37 °C. After incubation, Colony-Forming Units (CFU) were counted on PCA plates to quantify airborne microbial load. Bacterial identification was performed by MALDI-TOF MS (VITEK MS, bioMérieux, Marcy l’Etoile, France). A single colony from PCA culture was spotted onto a VITEK MS-DS target and overlaid with 1 μL of CHCA matrix (VITEK MS-CHCA; α-cyano-4-hydroxycinnamic acid), then air-dried for approximately 5 min. Mass spectra were acquired and processed in MYLA (bioMérieux) using routine pattern-matching against the reference database. Identifications meeting the manufacturer’s acceptance criteria (confidence ≥60%) were considered acceptable (Figure 1 and Figure 2).

### 2.5. Statistical Analysis

Airborne microbial contamination was expressed as CFU/m^3^. Since each SAS sampling run collected 1000 L (equivalent to 1 m^3^), colony counts on PCA plates correspond directly to CFU/m^3^. Data are reported as mean ± SD across the three IVI sessions. Given the repeated-measures design (two sampling locations measured repeatedly across seven time points within each IVI session), we performed a two-way repeated-measures ANOVA with location (procedure room air vs LAF field) and time point (T_−1_–T_5_) as within-session factors, including the location × time interaction. Post hoc within-session paired comparisons between locations at each time point were adjusted for multiple testing across the seven time points using the Holm method. Percent reduction was computed for each session and time point as (Room − LAF)/Room × 100 and is reported with 95% CI. A two-sided *p* value < 0.05 was considered statistically significant. Given the small number of sessions, results are interpreted as exploratory and emphasis is placed on effect sizes and CI. All analyses were performed using XLSTAT (Lumivero, 2024, Paris, France) and STATA software (release 16.1, StataCorp LLG, College Station, TX, USA, 2019).

## 3. Results

**Figure 3:**
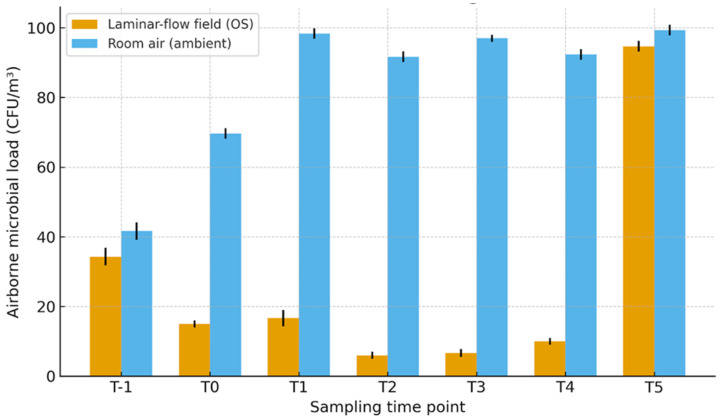
Airborne microbial load during IVI session.

Three intravitreal injection (IVI) sessions comprising approximately 20 injections per session were monitored and environmental samples were collected at different time points: T_−1_ (pre-session, flow off), T_0_–T_4_ (during activity, LAF on) and T_5_ (end of session, flow off). A total of 42 air samples (21 from the ambient clinical environment and 21 in proximity to the laminar-flow field) were analysed. When LAF is on (T_0_–T_4_), the mean microbial load in ambient clinic air was 89.8 ± 10.8 CFU/m^3^ (per-time values ≈ 68–99 CFU/m^3^) while in the laminar-flow field the mean was 10.9 ± 4.6 CFU/m^3^ (≈5–19 CFU/m^3^), corresponding to an average relative reduction of ≈ 88% (*p* < 0.01) (Figure 3). When the LAF device was on (T_0_–T_4_), airborne microbial load was significantly lower in the LAF field than in room air (repeated-measures ANOVA location effect *p* = 4.04 × 10^−5^; location × time *p* = 4.33 × 10^−16^; Table 1). Post hoc paired comparisons with Holm adjustment confirmed significant reductions at each on-time point (T_0_–T_4_). When the device was off (T_−1_ and T_5_), counts were similar between locations and no significant differences were observed after adjustment (Table 1).

Across the three IVI sessions (21 air samples per environment, corresponding to 21 m^3^ of sampled air in room air and 21 m^3^ under the laminar-flow field), a total of 2322 CFU were recovered, of which 1772 CFU (76.3%) originated from room air and 550 CFU (23.7%) from the laminar-flow field. The airborne microbiota was overwhelmingly composed of coagulase-negative staphylococci (CoNS), which accounted for 1825 CFU (78.7% of all colonies) when pooling both environments.

*Staphylococcus epidermidis* was the predominant species, representing 34.6% of all CFU (803/2322), with similar relative abundances in room air and under laminar flow (34.3% vs. 35.6%, respectively) (Table 2). Other frequent CoNS included *S. hominis* (12.5% overall), *S. haemolyticus* (10.3%), *S. warneri* (7.8%), *S. cohnii* (5.2%), *S. pettenkoferi* (4.5%), and *S. capitis* (3.9%). Among non-staphylococcal genera, *Micrococcus luteus* accounted for 14.9% of all CFU, while *Streptococcus gordonii* and *Moraxella osloensis* contributed 4.4% and 2.1%, respectively.

The relative species profiles were highly concordant between environments (Spearman *p* = 0.964; Bray–Curtis dissimilarity = 0.047, based on Table 2 relative abundances), indicating that LAF primarily reduced the overall microbial burden rather than changing the predominant species, with CoNS and *Micrococcus luteus* consistently dominating both environments. The main difference between the two settings was therefore quantitative rather than qualitative: the laminar-flow field exhibited a substantially lower overall burden of airborne microorganisms, but the microbiota profile remained typical of human skin and upper respiratory tract flora. No obligate pathogens such as *Staphylococcus aureus*, *Pseudomonas* spp., or *Enterobacterales* were isolated from any air sample. In addition, two airborne colonies of *Candida* spp. were detected as isolated fungal findings.

In total, 24 surface samples were obtained from the instrument-covering drape (3 sessions × 2 time points [mid-activity, T_2_, and end of activity, T_5_] × 4 media). Across all three days and all culture conditions, no bacterial or fungal growth was observed on any plate (0 CFU/plate), both at mid-session and at the end of the IVI activities. These findings indicate that, under the operating conditions evaluated, the instrument-covering drape remained microbiologically clean, supporting the effectiveness of field preparation, sterile material handling, and laminar-flow protection in limiting microbial deposition on critical procedural surfaces.

## 4. Discussion

The expansion of intravitreal therapies has led to an increasing procedural burden in outpatient ophthalmic settings, where maintaining adequate infection control standards is essential as patient volumes continue to rise [14]. The availability of intravitreal therapies has expanded dramatically in recent years, establishing them as first-line treatments for neovascular macular diseases and foveal macular edema [15]. Anti-VEGF agents have significantly improved visual outcomes in neovascular macular diseases and retinal vascular disorders, establishing intravitreal injection (IVI) as a cornerstone of retinal care. In selected cases, intravitreal corticosteroids remain an important therapeutic option, particularly when anti-VEGF response is insufficient or short-lived. As procedural demand increases globally in parallel with aging populations and higher diabetes prevalence, optimizing environmental safety during high-throughput IVI sessions is becoming increasingly relevant for healthcare systems.

Our study evaluated the effectiveness of a mobile laminar airflow (LAF) device in reducing microbial contamination during outpatient IVI sessions. By comparing the microbial load in the overall operating room with that in the perioperative field protected by LAF, we demonstrated that the implementation of a mobile laminar airflow (LAF) device significantly reduces airborne and surface microbial contamination in outpatient IVI settings. The observed reduction in colony-forming units (CFUs) in the protected operative field is consistent with prior microbiological investigations showing that LAF systems substantially decrease microbial burden at the surgical site compared with surrounding unprotected areas [16]. These results reinforce previous evidence indicating that ultraclean directed airflow effectively limits particulate dispersion during surgical activity [17].

Microbial contamination in operating environments is primarily driven by airborne particles and skin commensals dispersed by healthcare personnel, with bacterial load increasing in proportion to procedural duration and patient throughput when ultraclean airflow is absent [18,19,20]. In our setting, organisms such as *Staphylococcus epidermidis*, *Staphylococcus hominis*, and occasional fungal contaminants were detected predominantly in the general room environment, particularly toward the end of high-volume sessions. In contrast, the LAF-protected zone consistently maintained near-sterile conditions throughout the procedures.

A particularly relevant practical finding was the absence of microbial growth on the sterile filter drape covering the instrument area when protected by LAF. The persistence of 0 CFU contamination under these conditions represents a meaningful logistical advantage, as the drape maintained sterility for the entire duration of prolonged surgical lists. This eliminates the need for mid-session drape replacement and supports workflow efficiency in high-volume outpatient environments. The ability to preserve sterility of the instrument field without interruption has important implications for procedural standardization and resource optimization.

Published data by Scarpa et al. further support the capacity of mobile LAF systems to achieve ISO Class 5 ultraclean conditions over the operative field regardless of baseline room contamination levels [18]. Real-world clinical series have reported exceptionally low rates of post-IVI endophthalmitis in settings adopting LAF technology: a retrospective cohort of 6638 injections reported a single case (0.015%), whereas another series exceeding 10,000 injections reported no infections [12,21]. Although these rates compare favorably with those reported in standard operating theatres and office-based procedures (~0.007–0.03% and ~0.03–0.06%, respectively) [22], causal attribution cannot be established from environmental data alone. Rather, the reduction in microbial burden observed in our study provides mechanistic support for infection-control strategies that may contribute to lowering procedural risk.

Evidence from other surgical disciplines also highlights the protective role of LAF systems. In orthopedic surgery, LAF has been associated with approximately 36% reductions in bacterial contamination compared with non-LAF environments [23]. Similarly, outpatient cataract surgery performed in clean rooms equipped with mobile LAF units has been associated with zero postoperative infections in 1269 reported cases [24,25]. Collectively, these data suggest that the application of LAF technology represents a scalable environmental intervention capable of enhancing sterility assurance in high-volume procedural settings.

Overall, our findings indicate that mobile LAF systems effectively reduce microbial contamination in the operative field while maintaining sustained sterility of the protected instrument area throughout extended IVI sessions. The demonstration of persistent 0 CFU contamination on the sterile drape is particularly relevant from a practical standpoint, as it supports uninterrupted workflow and strengthens logistical efficiency without compromising infection-control standards.

### Limitations of the Study

This study has several limitations that should be considered when interpreting the findings. First, it was a single-centre investigation performed in one outpatient IVI room using one specific mobile LAF device; therefore, the results may not be directly generalizable to other institutions, room layouts, ventilation systems, or LAF technologies. Second, the number of monitored IVI sessions was limited, and although a dense sampling scheme across predefined time points was adopted, the study was not designed to capture seasonal variation or day-to-day fluctuations in background microbial load. Third, the endpoints were exclusively environmental; we quantified airborne and surface contamination but did not assess clinical outcomes such as post-injection endophthalmitis or other complications, so no direct causal inference can be made between the observed microbiological reductions and patient-level risk. Finally, adherence to aseptic technique, staff movements, and patient turnover were not formally quantified and may have contributed to the baseline contamination observed in room air; these factors are intrinsic to real-world practice and may differ across settings. These limitations underscore the need for larger, multicentre studies that combine standardized environmental monitoring with clinical surveillance of IVI-related infections to better define the role of mobile LAF within comprehensive infection-prevention strategies.

## 5. Conclusions

This study confirms that laminar airflow (LAF) systems, particularly the Operio Toul Mobile, represent an effective and reliable strategy for maintaining aseptic conditions during intravitreal injection (IVI) procedures in outpatient settings.

By maintaining air and surface sterility in the surgical field, even in high-volume outpatient workflows, LAF technology represents a practical and reliable adjunct to standard aseptic protocols, with the potential to further minimize the risk of post-injection endophthalmitis.

The consistent protection observed across multiple sampling points and surfaces highlights the robustness of LAF as a barrier against microbial spread, with particular effectiveness against skin commensals commonly implicated in post-IVI endophthalmitis. In addition to infection prevention, creating a safe microenvironment through a real-time sanitization process, the adoption of mobile LAF systems supports operational efficiency by enabling increased patient throughput and minimizing the time patients spend within the hospital environment.

Future research should focus on long-term multicenter evaluations to further validate these results, quantify the cost-effectiveness of LAF implementation, and explore its broader applicability across different ophthalmic and minor surgical procedures.

## Figures and Tables

**Table 1:** Microbial load at different time points and conditions.

Time Point	Procedure Room Air (CFU/m^3^)	LAF Field Air (CFU/m^3^)	Relative Reduction (%), Mean [95% CI]	*p*-Value (Holm-Adjusted)
T_−1_	41.7 ± 2.5	41.0 ± 1.0	1.45 [−7.60; 10.51]	>0.05
T_0_	69.7 ± 1.5	15.0 ± 1.0	78.44 [73.70; 83.18]	0.0021
T_1_	98.3 ± 1.5	16.7 ± 2.3	83.07 [77.67; 88.47]	0.0007
T_2_	91.7 ± 1.5	6.0 ± 1.0	93.46 [90.90; 96.02]	0.0004
T_3_	97.0 ± 1.0	6.7 ± 1.2	93.12 [89.99; 96.25]	0.0009
T_4_	92.3 ± 1.5	10.0 ± 1.0	89.16 [86.03; 92.28]	0.0012
T_5_	99.3 ± 1.5	98.7 ± 0.6	0.66 [−3.15; 4.46]	>0.05
T_0_–T_4_ (all LAF-on, session-level mean)	89.8 ± 0.7	10.9 ± 0.1	87.90 [87.34; 88.46]	0.000037

Mean (±SD) airborne microbial load (CFU/m^3^) in the laminar-flow field and ambient room air at each sampling time point during intravitreal injection sessions. At T_−1_ and T_5_, the room was at rest, whereas at all other sampling time points it was in routine use, with approximately four intravitreal injections performed per interval. Means (standard deviations) and significant differences between groups are given for 3 replicate measurements. *p*-values are Holm-adjusted for multiple comparisons across the seven time points and were obtained from within-session paired post hoc comparisons following a two-way repeated-measures ANOVA (location, time, and location × time). Relative reduction is reported as mean [95% CI].

**Table 2:** Microorganisms isolated from environmental air samples collected in room air and in the laminar-flow field during intravitreal injection sessions.

Microorganism	Room Air, Total CFU (%)	Laminar-Flow Field, Total CFU (%)	Total CFU (%)
*Staphylococcus epidermidis*	607 (34.3%)	196 (35.6%)	803 (34.6%)
*Micrococcus luteus*	267 (15.0%)	79 (14.4%)	346 (14.9%)
*Staphylococcus hominis*	219 (12.4%)	70 (12.7%)	289 (12.4%)
*Staphylococcus haemolyticus*	190 (10.7%)	48 (8.7%)	238 (10.3%)
*Staphylococcus warneri*	135 (7.6%)	46 (8.4%)	181 (7.8%)
*Staphylococcus cohnii*	94 (5.3%)	26 (4.7%)	120 (5.2%)
*Staphylococcus pettenkoferi*	83 (4.7%)	21 (3.8%)	104 (4.5%)
*Streptococcus gordonii*	79 (4.5%)	22 (4.0%)	101 (4.4%)
*Staphylococcus capitis*	66 (3.7%)	24 (4.4%)	90 (3.9%)
*Moraxella osloensis*	30 (1.7%)	18 (3.3%)	48 (2.1%)
*Candida* spp.	2 (0.1%)	0 (0%)	2 (0.1%)

CFU were calculated from 21 samples per environment (3 sessions × 7 time points; 1 m^3^ per sample). Coagulase-negative staphylococci (CoNS: *S. epidermidis*, *S. hominis*, *S. haemolyticus*, *S. warneri*, *S. cohnii*, *S. capitis*, *S. pettenkoferi*) accounted for ~78.7% of all CFU (1825/2322). All identified species belong to commensal skin and upper respiratory/oral flora; no obligate pathogens (e.g., *S. aureus*, *Pseudomonas* spp., *Enterobacterales*) were detected.

## Data Availability

All data were included in the manuscript.
